# A Simple Picaxe Microcontroller Pulse Source for Juxtacellular Neuronal Labelling †

**DOI:** 10.3390/bioengineering3040027

**Published:** 2016-10-19

**Authors:** Anthony J. M. Verberne

**Affiliations:** Clinical Pharmacology and Therapeutics Unit, Department of Medicine, University of Melbourne, Austin Health, Heidelberg Victoria, Heidelberg 3084, Australia; antonius@unimelb.edu.au; Tel.: +61-3-9496-5878; Fax: +61-3-9459-3510

**Keywords:** juxtacellular, Picaxe, neurophysiology, neuron, microcontroller

## Abstract

Juxtacellular neuronal labelling is a method which allows neurophysiologists to fill physiologically-identified neurons with small positively-charged marker molecules. Labelled neurons are identified by histochemical processing of brain sections along with immunohistochemical identification of neuropeptides, neurotransmitters, neurotransmitter transporters or biosynthetic enzymes. A microcontroller-based pulser circuit and associated BASIC software script is described for incorporation into the design of a commercially-available intracellular electrometer for use in juxtacellular neuronal labelling. Printed circuit board construction has been used for reliability and reproducibility. The current design obviates the need for a separate digital pulse source and simplifies the juxtacellular neuronal labelling procedure.

## 1. Introduction

Juxtacellular neuronal labelling has become an important addition to the tool box of the in vivo electrophysiologist. First described by Pinault [1,2], juxtacellular neuronal labelling has enabled electrophysiologists to fill single neurons with a tracer molecule in order to determine the precise location of the neuron and also to identify its neurochemical identity. Prior to the development of juxtacellular neuronal labelling, labelling of single neurons could only be achieved by using the vastly more difficult technique of in vivo intracellular recording [3].

Juxtacellular neuronal labelling involves recording the extracellular activity of single neurons using a glass microelectrode filled with a solution containing a biotin derivative, such as biotinamide (Neurobiotin) dissolved in 0.5 M sodium acetate. Note that, in principle, other small molecules may also be used to label neurons as long as they are positively charged in solution. Once a suitable neuron has been characterised, positive-going extracellular current pulses (0–10 nA) are applied through the amplifier headstage at a gradually increasing amplitude. If the tip of the electrode is sufficiently close to the neuron, the discharge of the neuron will become entrained to the current pulses. The duration of the entrainment period, as well as the current intensity required to produce entrainment, determine the intensity and completeness of the labelling since the biotinamide is transported intracellularly during entrainment. Subsequent histological and histochemical processing is used to identify the labelled neuron [2,4,5,6]. The mechanism that underpins juxtacellular neuronal labelling is uncertain but may be related to previous observations that extracellular current applied close to the neuronal membrane influences neuronal excitability [7].

The impedance of the recording microelectrode should be in the order of 20 MΩ or higher. This value is higher than the electrode impedances used in conventional extracellular neuronal recording (5–10 MΩ). High impedance electrodes do not “see” as far as low impedance electrodes and so need to be closer to a neuron compared to microelectrodes with lower impedances. As a result, large amplitude spikes are recorded.

The equipment required for juxtacellular neuronal labelling differs from that used for extracellular electrophysiological recording because the amplifier must have a headstage that is capable of passing current pulses through the recording microelectrode. Pinault [2] found that 200 ms pulses delivered at a 50% duty cycle (2.5 Hz) reliably entrained the neuronal discharge rate. The pulse rate and duration are controlled by a pulse source which is the subject of this article.

## 2. Experimental Section

### 2.1. Overall Design

In the present design, a Picaxe 14M2 microcontroller-based pulse generation circuit is described and this been incorporated into the case of a commercially-available intracellular electrometer suitable for use in juxtacellular neuronal labelling. While the present design is based on a Picaxe chip, other microcontrollers could also be used. However, the Picaxe option was chosen because of low cost, free programming software, and the ability to program the chip in BASIC [8]. The design also has a “Search” function which uses 1 nA 200 ms pulses to monitor the electrode impedance while searching for a neuron as described previously [9]. In addition, we have incorporated a feature which allows application of pulses with a duration of between 100 and 800 ms every 5 seconds to allow examination of the relationship between pulse width and the number of evoked spikes for the neuron under test.

**Figure 1:**
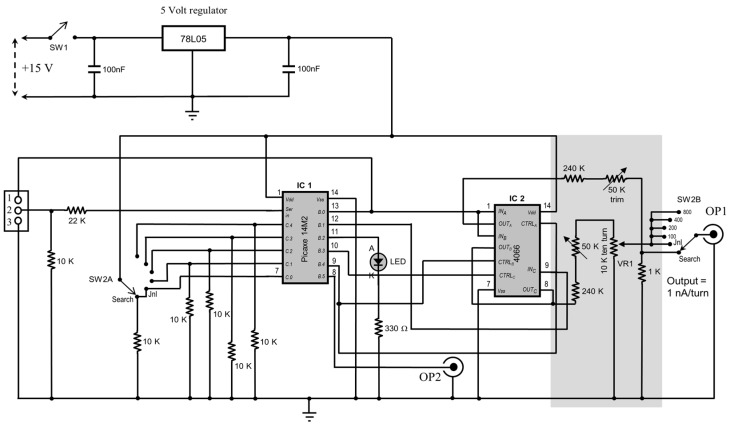
Schematic of the Picaxe microcontroller pulse generator. Main components include IC1 (Picaxe 14M2 microcontroller) and IC2 (CD4066 quad bilateral switch). SW2A and SW2B (function selector switch) determine the pulser output. The 50 K trim potentiometers are used to calibrate the output voltage.

Microcontrollers are microchips that store and implement BASIC computer programs using built-in flash memory. Here, a simple and cheap microcontroller program and circuit is described. This device provides the 2.5 Hz, 50% duty cycle pulse output that can be used to control the current pulses required for juxtacellular neuronal labelling. We used an intracellular electrometer (Model 757; World Precision Instruments, Sarasota, FL, USA) which requires a 20 mV pulse amplitude input in order to pass a pulse of 1 nA intensity through the electrode irrespective of electrode impedance. The circuit is easily built from standard parts (see Figure 1). The Picaxe 14M2 chip can be obtained from several suppliers (e.g., Wiltronics Ltd, Alfredton, Australia [10]) and free programming software can be obtained from the Picaxe website [11].

Since our laboratory uses WPI Intra 757 electrometers (World Precision Instruments, Sarasota, FL, USA), a modification of this device is described. However, the circuit could easily be adapted for use with other electrometer designs. For example, the A-M Systems Model 3100 (A-M Systems, Carlsborg, WA, USA)) and iWorx ETH 3100 Intracellular Electrometers (iWorx, Dover, NH, USA) both require a 10 mV pulse amplitude input in order to pass a pulse of 1 nA intensity through the electrode. This requirement can be met by changing the 240 k resistors in both the voltage divider circuits to 470 k, 1% resistors.

**Figure 2:**
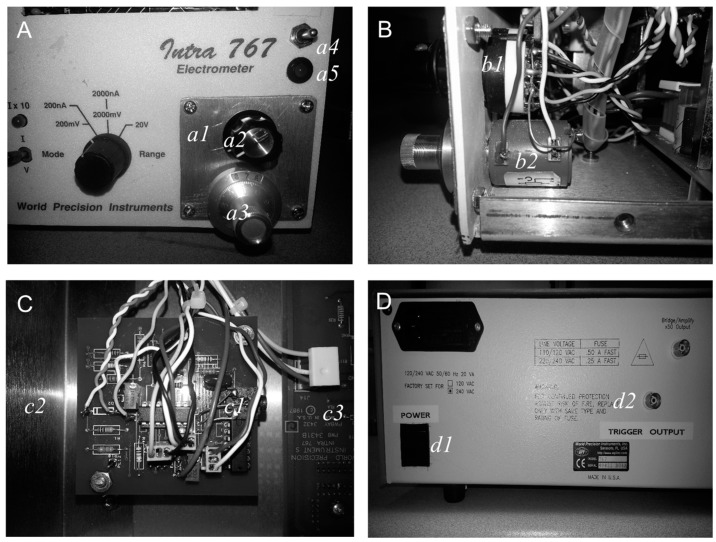
WPI electrometer panel modifications. (**A**), The front panel power switch has been removed and fixed to the back panel of the instrument. In its place is an aluminium panel (*a1*) onto which is fixed the 10-turn (10k) potentiometer (*a2*) and 6-position function switch (SW2A,B; *a3*). The power switch (SW1; *a4*) and the LED monitor (*a5*) are mounted to the right of the panel. (**B**), Side-on view of the 10-turn potentiometer (*b1*) and the function switch (*b3*). (**C**), Pulser printed circuit board (PCB) (*c1*) mounted inside the WPI Intra 767 case between power supply section at left (*c2*; not shown) and main circuit board (*c3*). Note that this is a working prototype but is not the final PCB design available in the Supplementary files. (**D**), Rear panel showing new location of main power switch (*d1*) and trigger output connector (*d2*). LED = Light Emitting Diode.

Behind the front panel of the Intra 757 is a printed circuit board on which are mounted the controls that appear on the front panel of the instrument. The only available “free” space on the front panel for addition of the power switch (SW1), function selector switch (SW2A & 2B), monitor LED (light emitting diode) and the current pulse amplitude control (VR1) is at the position of the main power switch of the Intra 767. Therefore, the power switch was relocated to the back panel. A piece of aluminium panel was fixed into place over the gap left after removal of the power switch. This was then used to fix the controls (VR1 and SW2) required for the pulser circuit while the monitor LED and power switch (SW1) are located above this panel (see Figure 2).

### 2.2. Circuit Design

**Figure 3:**
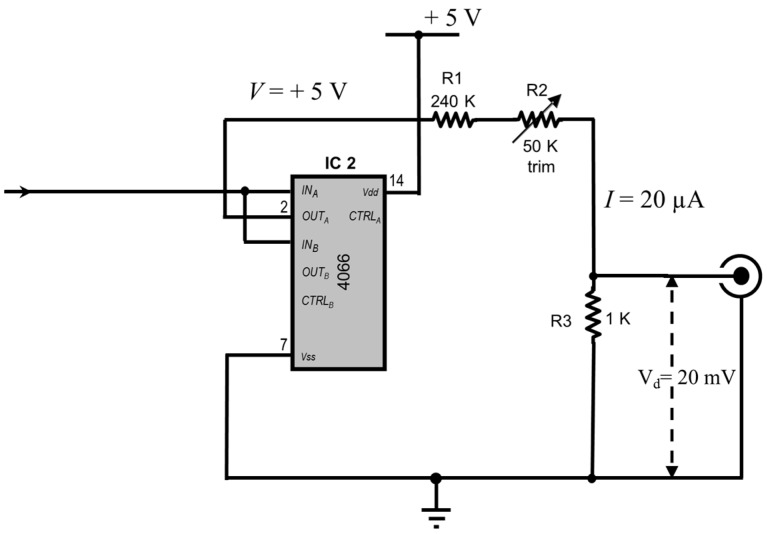
Voltage divider circuit operation. The outputs of the CD4066 chip are fed to a voltage divider consisting of a 240 K fixed resistor, a 50 K 25-turn trim potentiometer and a fixed 1 K resistor. A total resistance of 250 K will result in a current of *I* = 5/250,000 = 20 µA flowing through the voltage divider. This will result in a fixed potential of *V_d_* = 20 × 1000 = 20 mV. The WPI Intra 767 will produce current pulses of 1 nA for a 20 mV input.

The pulse generation circuit is based around IC1, a Picaxe 14M2 microcontroller chip (Figure 1). The 14M2 chip must be programmed (see Supplementary files) after installation onto the circuit board (see Section 2.5. Programming the 14M2 chip). The circuit is powered by a 5 V regulated supply which is produced by the LM78L05 (IC 3) regulator chip. Power is taken from the topmost power supply board of the Intra 767 at the connector marked “J2”. This connector has three pins and +15 V appears between “V+” and the centre connector (ground). The +15 V power rail is converted to +5 V by the 3-terminal regulator IC3 (LM78L05). Output of the pulser circuit is taken from the wiper of SW2B and is connected to the “Stimulus Input” of the Intra 767 front panel circuit board (actual connection to the circuit board). In addition, OP2 is connected to a socket on the rear panel of the Intra767 (Figure 3). A bill of materials is listed in Table 1.

Pin 2 of IC1 is used to load the program onto the 14M2 chip. Pins 3–7 are used as inputs to control which section of the BASIC code is run. These inputs are all kept “low” by tying them to ground via 10 k resistors. Setting pins 3–7 (inputs C.0–C.5) “high” is achieved by connecting them to the 5 V supply rail. Rotary switch SW2A selects the various functions that have been incorporated into the design.

### 2.3. Code Operation

The BASIC program is composed of several subroutines starting with “main”. The “main” routine first defines pins B.0–B.5 as output pins on the Picaxe chip. It then detects the state of pins C.0 to C.4. If a high state is detected on pinC.0 (pinC.0 = 1) then the program jumps to the “do-JNL” subroutine. Before execution of the selected subroutine the state of pins C.0 to C.4 is re-checked. This has been included so that if the position of SW2 is changed during operation, the program jumps to the new subroutine. In each subroutine there is a line (pulsout B.5, 500) which sets a 5 ms pulse at output B.5 (pin 8 of the Picaxe 14M2 IC). This is used to trigger the sweep of the oscilloscope while monitoring the entrainment of the neuron to the juxtacellular current pulses. It is accessed at OP2 which is connected to a BNC socket on the rear panel of the electrometer amplifier.

Switching to the “Search” position connects pin 7 (C.0) of the 14M2 chip to the 5 V supply rail and sets this input “high”. As a result, a high state is detected at C.0 causing the “main” subroutine to jump to the “do_JNL” subroutine. The “do_JNL” subroutine sets pin 13 and 11 (outputs B.0 and B.1) high for 200 ms. This turns on the LED for 200 ms and, via the output of switch A of IC2 (pin 2), connects the output to the voltage divider consisting of the 240 k, the 50 k trimmer and the 1 k resistor. This sets the amplitude of the output pulse to 20 mV.

Note the following calculation with reference to Figure 3

If *I* is the current flowing through the voltage divider, *V* (= +5 V) is the voltage applied to the voltage divider, and *R* is the total resistance (R1 + R2 + R3) which should total 250 K by adjustment of the 50 k trimmer, then *I* = 5/250,000 = 20 μA.

The voltage drop, *V*_d_ across the 1 k is then *I* × 1000 = 20 mV which sets the current passed through the Intra 767 headstage to 1 nA.

Switch SW1 is mounted onto the front panel along with a light emitting diode (used as an indicator lamp) and a 10-turn potentiometer that controls the current pulse intensity. The 10-turn current intensity potentiometer allows application of current pulses of increasing intensity while awaiting entrainment of the discharge of the neuron. The “search” function (1 nA pulses) uses separate output circuitry in which the 1 nA current is set using a fixed 1 kΩ resistor. IC2 is a quad bilateral CMOS (4066) switch that switches the output pulses from IC1 to the respective voltage-divider output circuits. The first voltage divider circuit consists of a 240 K fixed resistor, a 50 K trim pot and a 10 K 10-turn potentiometer. The latter is used to dial up current pulse intensity from 0 to10 nA. The second voltage divider (240 K, 50 K trim pot, 1 K resistor) is used to derive a fixed voltage of 20 mV corresponding to 1 nA pulses used as a search stimulus. The entire circuit was built on a printed circuit board (PCB) designed by the author using PCB design freeware (DesignSpark; RS Electronics Ltd, Karnataka, India). The author recommends using IC sockets in case of failure of ICs 1 or 2.

### 2.4. Calibration

In order for the device to match the “Stimulus Input” requirements of the Intra 757, there are two trim potentiometers in the output circuitry voltage dividers that require adjustment prior to use. When SW1 is set to the “Search” option, the voltage that appears across the 1 k resistor (and the output at OP) should be 20 mV. This should be checked by connecting an oscilloscope across the output of the pulser circuit once the PCB has been populated with all of the electronic components and the BASIC script has been downloaded to the microcontroller chip. The 10 k trimmer should be adjusted until exactly 20 mV is measured across the 1 k resistor. Similarly, when SW2 is set to “JNL”, 20 mV should appear at the output when the 10-turn potentiometer is set to “1 nA”. Once the trimpots have been adjusted, a small dot of sealing wax should be dropped onto the adjustment screw to fix it in place.

### 2.5. Programming the 14M2 Chip

The Picaxe 14M2 has a “Serial In” input. The pulser printed circuit board includes a stereo phono socket which is used to programme the 14M2 chip via the Serial In (connection see Figure 1). While the 14M2 can be programmed using a PC serial port, it is most easily done using a PC USB port and using a Picaxe USB cable (AXE027). This cable converts the USB output from the PC on which the program resides to a serial data form which the Picaxe chip can accept.

Using the Picaxe program editor (Picaxe Program version 6) [11] enter the BASIC code listed in the Supplementary file section (PicaxeStim.txt). After completion of construction of the PCB and all components have been mounted correctly, connect a 15 VDC power supply to the pins marked pins 11 (positive) and 1 on the PCB. Connect the AXE27 USB cable to a USB port and allow the USB driver to install. Then insert the phono plug into the phono socket on the PCB and switch on the power supply. Select the “download” option in the program editor and program should download onto the 14M2 chip. The LED should begin to flash.

### 2.6. Operation

Incorporation of the Picaxe pulser circuit into the case of the intracellular amplifier simplifies the number of external connections that need to be made to the electrometer. However, the intracellular amplifier used in this design has limited gain and limited bandpass filtering. In order to obtain bandpass filtration and additional gain we use an additional standard extracellular amplifier whose output is connected to an oscilloscope and a computerised data acquisition system. In addition, in order to synchronise the pulser output with the sweep of the oscilloscope used to monitor the juxtacellular labelling process, a 5 ms pulse output is provided at OP2. This consists of a BNC socket mounted on the rear panel of the Intra 767.

In normal operation, the brain area of interest is identified using stereotaxic coordinates combined with other physiological “landmarks”. Once in the vicinity of the target area, the selector switch SW2 is set to “Search” and then the device is powered up by closing SW1. The panel LED should begin to flash and 1 nA current pulses are applied to the electrode. The electrode impedance can be monitored by noting the amplitude of the output of the bridge amplifier.

## 3. Conclusion

A microcontroller-based pulse generation circuit is described which can be used as a pulse source during juxtacellular neuronal labelling. The design is simple to construct owing to PCB-based construction and is readily incorporated into the design of existing neurophysiological amplifiers. Juxtacellular neuronal labelling is of increasing importance to neurophysiologist interested in neuronal chemistry and neurotransmitters of physiologically-identified neurons.

## Figures and Tables

**Table 1:** Bill of components.

Semiconductors	Picaxe 14M2 Microcontroller Chip LM78L05 Low Power 3 Terminal 5 V Regulator Light Emitting Diode (A standard red or green led is compatible with the 330 ω resistor used in the prototype. If a blue or white led is chosen, the value of the resistor will need to be adjusted.)
Resistors	240 K, 1% × 2 22 K, 1% 10 K, 1% × 6 1 K, 1% 330 Ω, 1% 10 K Helical 10-turn Potentiometer 50 K 10-turn trim potentiometer × 2
Capacitors	100 nF Polyester × 2
Hardware	14 pin DIP IC Socket × 2 2 pole, 6 Position Switch Stereo Phono Socket 3.5 mm, PCB Mount Printed Circuit Board (See Text for Details) Picaxe AXE027 USB Cable BNC Socket

**Notes:** A printed circuit board design can be obtained from the author. This design can be submitted to a printed circuit board (PCB) manufacturer who does small production runs.

## Supplementary Materials

The following are available online at http://www.mdpi.com/2306-5354/3/4/27/s1. Figure: Printed circuit board, Txt: PicaxeStim.txt.
