# Supplementary figures and images for: A Simple Picaxe Microcontroller Pulse Source for Juxtacellular Neuronal Labelling
Source: Bioengineering (Basel). 2016 Oct 19;3(4):27. doi: 10.3390/bioengineering3040027 (PMC5597270; doi:10.3390/bioengineering3040027)

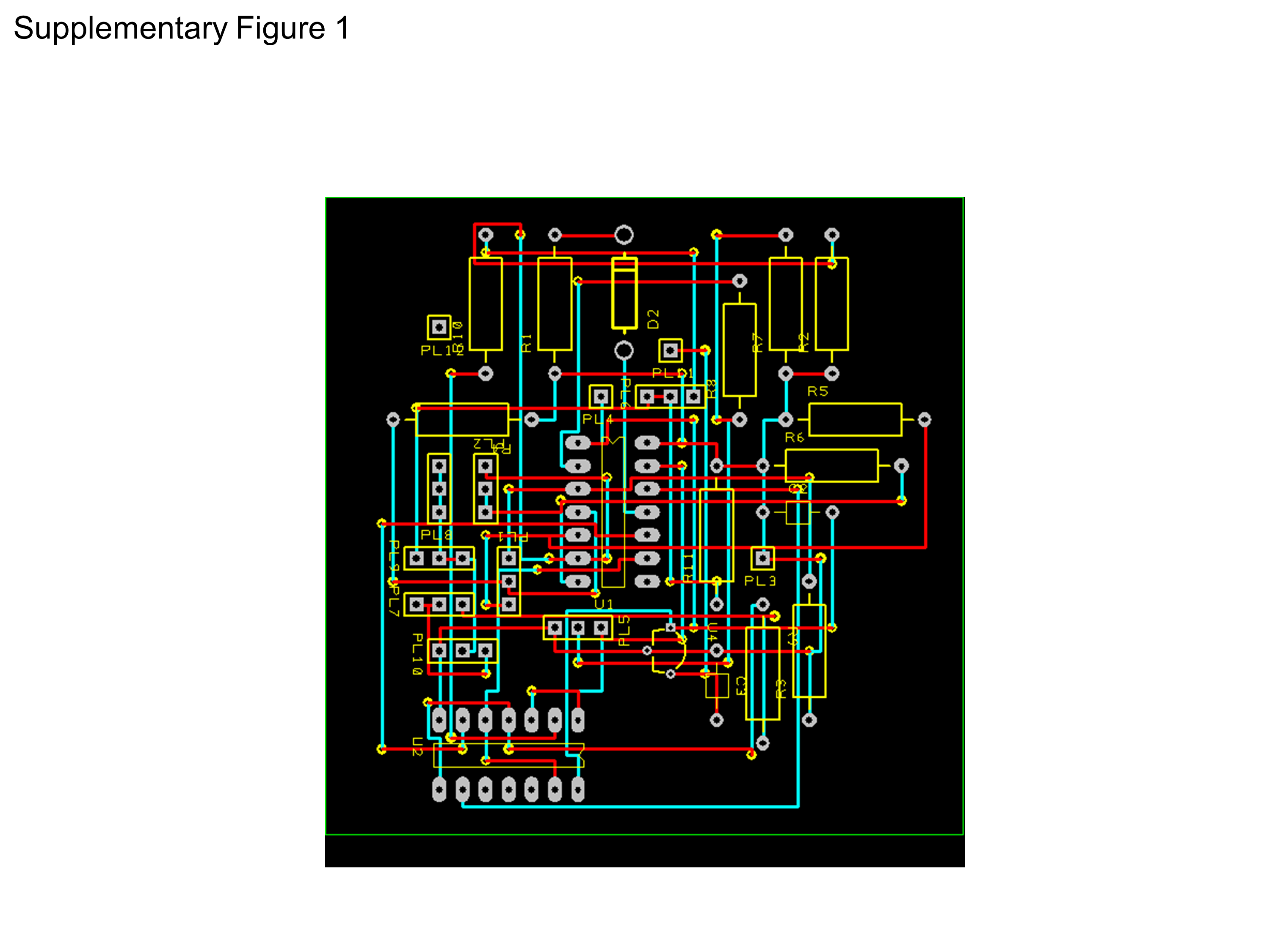

Supplement: Supplementary file 1 [file bioengineering-03-00027-s001.zip › bioengineering-152012-final-supplementary/Supplementary Figure 1.tif]
